# Aw, snap! How reversible protein lipidation helps plants deal with sudden temperature dives

**DOI:** 10.1093/plcell/koae118

**Published:** 2024-04-12

**Authors:** Vicky Howe

**Affiliations:** Assistant Features Editor, The Plant Cell, American Society of Plant Biologists; Department of Developmental Genetics, Heinrich-Heine University, Düsseldorf 40225, Germany

Coming from a country of evergreen vegetation, I am fascinated by the daily changes occurring when spring finally arrives here in Germany after a leafless winter. Each year, I notice that after a few warm days in early spring, the flowers start blooming and green shoots erupt from the earth. Inevitably, though, the cold returns, and the tender new growth is exposed to frosts and freezing temperatures. Yet the plants live on. How do plants achieve such resilience to abrupt temperature plummets? Despite being an active area of research, many of the underlying molecular mechanisms regulating cold stress responses in plants remain unknown.

This study by **Qinyi Ye, Lihua Zheng, and colleagues** (Ye et al. 2024) may have uncovered some vital clues. The group studied a transcription factor in the model legume species *Medicago truncatula*, MtNAC80, which they found to be highly expressed in response to acute cold exposure. Interestingly, under normal conditions, MtNAC80 is primarily located at the plasma membrane. It translocates to the nucleus to activate expression of its target genes only upon sudden cold stress. As MtNAC80 lacks any transmembrane domain, the group searched for possible post-translational modifications that could provide membrane anchorage. They found that MtNAC80 undergoes S-acylation, also known as S-palmitoylation, which involves the attachment of a lipophilic fatty acid to a specific cysteine residue by a protein S-acyltransferase (PAT). This allows the protein to associate with the hydrophobic cell membranes. Importantly, S-acylation is reversible, with de-acylation being catalyzed by acyl protein thioesterases (APTs) (see Figure).

**Figure.**
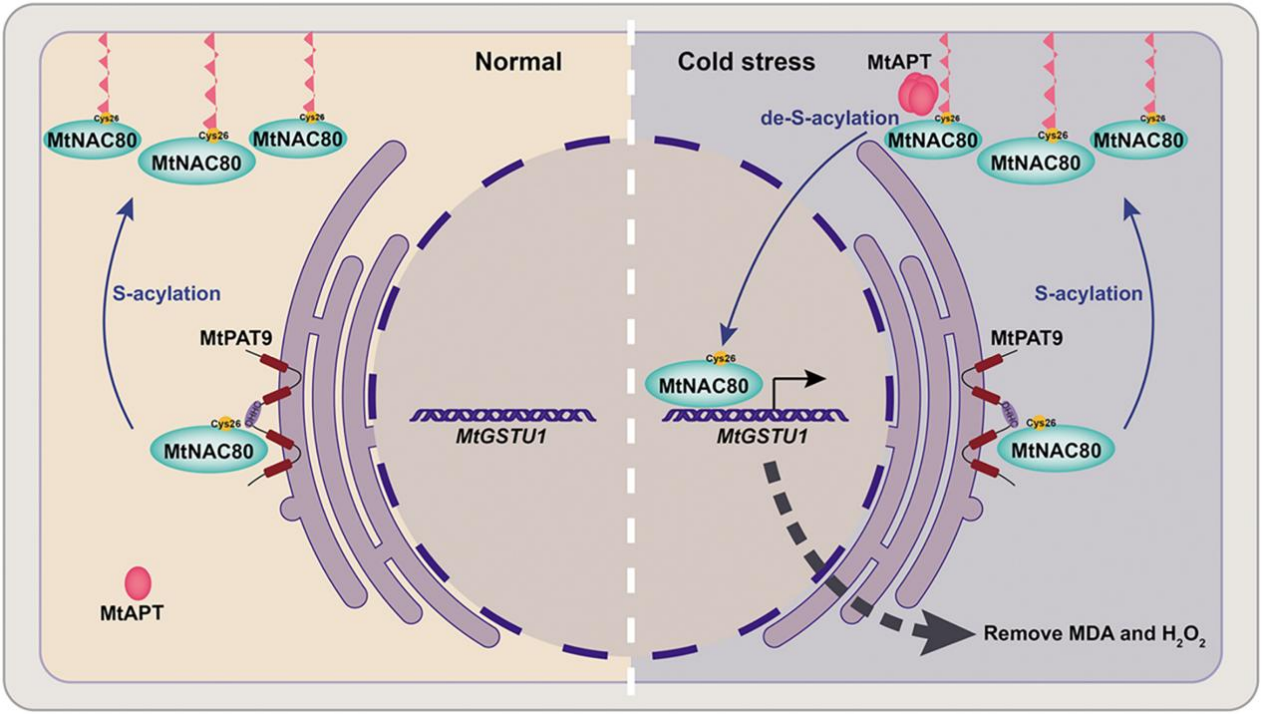
Sudden cold stress triggers de-S-acylation of the transcription factor MtNAC80 by an acyl protein thioesterase (MtAPT), removing MtNAC80's lipid anchor and allowing it to translocate from the plasma membrane to the nucleus. Here MtNAC80 upregulates expression of genes such as *MtGSTU1*, which reduce levels of reactive oxygen species like MDA and H_2_O_2_, protecting the plant from cold-induced oxidative damage. S-acylation at Cys26 by the protein S-acyltransferase MtPAT9 returns MtNAC80 to the plasma membrane, where it resides under normal conditions. Adapted from Ye et al. (2024), Figure 9.

Naturally, the group wanted to know which proteins were responsible for the acylation and de-acylation of MtNAC80 in response to cold stress. Of 24 potential MtPATs in the *M. truncatula* genome, one, MtPAT9, specifically interacted with MtNAC80. Furthermore, MtNAC80-GFP expressed in *mtpat9* knockouts had reduced S-acylation levels and a cytoplasmic localization, confirming that MtPAT9 is required for the S-acylation and membrane localization of MtNAC80. The group similarly identified a thioesterase, MtAPT1, which could de-acylate MtNAC80. Moreover, they showed that MtAPT1 was activated upon cold stress, rapidly transitioning from its inactive monomer state to an active tetramer. This provides a mechanism by which cold stress triggers the de-acylation of MtNAC80 by MtAPT1, enabling MtNAC80 to translocate to the nucleus and activate transcription of its target genes (see Figure).

To identify these target genes, Ye and colleagues looked for genes with lower expression levels in *mtnac80* mutants compared to wild type and found many were involved in reducing oxidative stress. One of these genes, the glutathione S-transferase (GST) *MtGSTU1*, was in fact upregulated by MtNAC80 under cold stress conditions, reducing the levels of cold-induced reactive oxygen species. Additionally, they found that disrupting the S-acylation/-de-S-acylation cycle of MtNAC80 by knocking out either *MtPAT9* or *MtAPT1* resulted in impaired GST activity and increased reactive oxygen species under cold stress. Notably, *mtpat9* mutants exhibited much poorer freezing tolerance compared to wild-type plants.

Taken together, this study has uncovered a novel role of S-acylation and de-S-acylation in promoting freezing tolerance in *M. truncatula* by preventing oxidative stress induced by abrupt temperature drops. Moreover, because both PATs and APTs appear to be highly promiscuous, the S-acylation cycle could provide a general mechanism for regulating diverse stress responses, including drought or salt stress (Zhou et al. 2013; Duan et al. 2017). Importantly, understanding mechanisms to cope with abrupt temperature drops has implications for directing crop improvement strategies in areas prone to late spring cold snaps.
